# Sick leave absence and the relationship between intra-generational social mobility and mortality: health selection in Sweden

**DOI:** 10.1186/s12889-019-8103-4

**Published:** 2020-01-06

**Authors:** Sunnee Billingsley

**Affiliations:** Department of Sociology and Demography Unit, Stockholm University, S-106 91 Stockholm, Sweden

## Abstract

**Background:**

Poor health could influence how individuals are sorted into occupational classes. Health selection has therefore been considered a potential modifier to the mortality class gradient through differences in social mobility. Direct health selection in particular may operate in the short-term as poor health may lead to reduced work hours or achievement, downward social mobility, unemployment or restricted upward mobility, and death. In this study, the relationship between social mobility and mortality (all-cause, cancer-related, cardiovascular disease-related (CVD), and suicide) is explored when the relationship is adjusted for poor health.

**Methods:**

Using Swedish register data (1996–2012) and discrete time event-history analysis, odds ratios and average marginal effects (AME) of social mobility and unemployment on mortality are observed before and after accounting for sickness absence in the previous year.

**Results:**

After adjusting for sickness absence, all-cause mortality remained lower for men after upward mobility in comparison to not being mobile (OR 0.82, AME -0.0003, CI − 0.0003 to − 0.0002). Similarly, upward mobility continued to be associated with lower cancer-related mortality for men (OR 0.85, AME -0.00008, CI − 0.00002 to − 0.0002), CVD-related mortality for men (OR 0.76, AME -0.0001, CI − 0.00006 to − 0.0002) and suicide for women (OR 0.67, AME -0.00002, CI − 0.000002 to − 0.00003). The relationship between unemployment and mortality also persisted across most causes of death for both men and women after controlling for previous sickness absence. In contrast, adjusting for sickness absence renders the relationship between downward mobility and cancer-related mortality not statistically different from the non-mobile.

**Conclusions:**

Health selection plays a role in how downward mobility is linked to cancer related deaths. It additionally accounts for a portion of why upward mobility is associated with lower mortality. That health selection plays a role in how social mobility and mortality are related may be unexpected in a context with strong job protection. Job protection does not, however, equalize opportunities for upward mobility, which may be limited for those who have been ill. Because intra-generational upward mobility and mortality remained related after adjusting for sickness absence, other important mechanisms such as indirect selection or social causation should be explored.

## Background

Relative inequalities in mortality by level of social class remain and have even increased in Europe in recent decades, including in Sweden [1]; individuals in higher social classes tend to live longer than those further down in the social hierarchy. Health selection has been considered a potential modifier to this mortality gradient through differences in social mobility; the health selection hypothesis predicts that individuals are sorted into classes on the basis of their health [2, 3]. Upward intra-generational mobility—changing a job to one that is in a higher occupational class—may not be accessible to all if opportunities are restricted by factors such as poor health. Additionally, poor health may lead to downward social mobility—taking a job in a lower social class. This hypothesis is therefore predicated on health being a determining factor of social mobility. But findings are inconclusive on whether this is the case. Much research suggests that social mobility is not associated with previous health [4–11], whereas some findings indicate a modest effect of health on social mobility [12–17], on financial deprivation [18] and the attainment of supervisory/managerial positions and income change [19].

In studies on the association between social mobility and mortality, the usual research design may have minimized the role of health selection. In Swedish research in particular [20–23], mobility is measured by a difference in social class at two time points that are years apart (generally 5 to 15 years) with a mortality follow-up period beginning after the second measure of class. The longer the interval between measures, the more studies may suffer from a “healthy survivor effect”; individuals who survive until the second measure are healthier than others. Moreover, mobility events that occur during the mortality follow-up period are not observed in these studies. Such an analytical design captures long-term relationships between social mobility and mortality, which are suitable for causal or indirect health selection mechanisms that operate over a long window of time [2, 4, 7]. In contrast, direct health selection may operate more in the short-term as poor health may immediately lead to reduced work hours or achievement, downward social mobility or no upward mobility [18, 24], and death. Occupational class measures must therefore be observed continually and at close intervals. In a recent application of this approach, intra-generational upward mobility was particularly linked to lower mortality risk across a wide range of causes of death for men, net of origin and destination class associations [25]. The current study builds on this preliminary support for the idea that health selection plays a role in how mobility and mortality are linked in Sweden by examining the contribution of health selection to this relationship more directly.

The aim of this study is to examine whether accounting for health status in the previous year weakens the short-term relationship between intra-generational social mobility and premature mortality. Standard measures in the literature for measuring health have included self-reported measures of poor health (physical and psychiatric), cardiometabolic factors, or medical records such as hospital admission. Health is measured in the current study by whether an individual took sick leave from work, referred to as sickness absence. This measure is objective, covers a broad spectrum of severe chronic diseases and disorders, as well as illnesses that might not be included in hospitalization records because of treatment at out-patient clinics. Sickness absence in Sweden has been changing with an increasing trend of psychiatric disorders (stress, in particular), reaching around 35% of all sickness absence in 2014 and a decreasing trend in musculoskeletal disorders, falling to 27% by 2014 [26]. Taking sick leave has been linked to negative earnings and career trajectories [27], as well as to all-cause mortality, cancer and CVD-related mortality and suicide, including psychiatric-related sickness absence [28–31].

These specific causes of death—all-cause mortality, cancer-related mortality, CVD-related mortality and suicide— are analyzed separately in this study. Over the time period of this study (1997–2012), CVD-related causes of death were most prominent for both men and women, followed by cancer [32]. External causes of death were also an important contributor, but these causes of death have not been found to be related to social mobility in Sweden [25]. Health selection is likely to be more present in mortality due to chronic [33] rather than acute diseases or disorders. All three causes of death here can be the outcome of conditions labeled as chronic [32], and there is great variation within the categories of cancer, mental disorders and cardiovascular disease. However, it may be that both cancer-related deaths and suicide are more likely preceded by periods of physical or mental illness that greatly reduce work capacity and impact career trajectories than cardiovascular disease.

If poor health leads to sickness absence and restricts access to upward mobility or increases incidence of downward mobility, the relationships between social mobility and mortality should be weakened when accounting for sickness absence. This hypothesis should be particularly relevant for cancer, where sick leave would be taken on the basis of poor health related to these diseases, as well as to suicide, where sick leave would presumably be taken for psychiatric disorders such as depression. However, the relationship between sickness absence and cause of death is not necessarily straightforward; for example, higher suicide risk was established for individuals in Sweden who had also taken somatic sickness absence, such as for musculoskeletal and digestive diseases [28]. CVD-related mortality encompasses major sudden causes of death (such as massive stroke, fatal arrhythmias, acute myocardial infarction, massive pulmonary embolism and acute aortic catastrophe), which may lessen the role that health selection plays in social mobility because there is less time for illness to influence careers.

The pathway leading from poor health to downward mobility may entail either a preference for a different set of circumstances that are potentially less demanding or that an individual who has lost their position was not able to obtain a new job in the same occupational class. An alternative labor market consequence is that an individual is not able to find new employment and is unemployed. This study therefore additionally explores unemployment as a potential extension of downward mobility [34]. Whether the health selection hypothesis has merit may depend on the country or social policy context being examined [24, 35]. In countries with a well-developed social benefits system and job protection policies such as Sweden [36, 37], individuals should be protected from losing their job if a physical or psychiatric disorder leads to reduced work ability. The Swedish Law on employment states that a dismissal of employment cannot be made without objective reasons that do not include poor health. In addition, the employer is obliged to help the previously ill worker return to work, and if necessary also offer a new job at the workplace. Sick leave benefits have maintained a replacement rate of 75 to 80% of an individual’s wage (up to a maximum income level) over the last decades, which supports those in ill health to take a leave of absence and maintain a similar standard of living. Sickness absence is also provided with flexibility, whereby individuals can take sickness benefits that amount to 25, 50, 75% or 100% of their work time, depending on the extent of reduced work ability. These provisions, including the anti-discrimination legislation related to sick workers, should minimize direct health selection in the Swedish context [24]. In relation to health selection into upward mobility, however, these provisions are not likely to moderate how promotion possibilities are limited following sickness absence, which has been demonstrated in the US [38].

## Methods

### Register data

This study is based on Swedish register data that provides basic demographic information, socio-economic variables, sickness absence and mortality timing from population-based registers collected by Statistics Sweden. All-cause mortality was considered as well as specific causes of death delineated according to ICD-10 coding: deaths due to all cancers (C00-C97, D00-D48), CVD-related mortality (I00-I99) and suicide, including undetermined causes of death (X60-X84, Y10-Y34). Annual occupational information was provided over the time period 1996–2012 (in October/November each year). Complete coverage of occupational information was provided for all individuals who work in the public sector, and all those working in private companies with 500 or more employees. Information on individuals working in smaller private firms was restricted to a random selection each year in which the chance of being included in the register declined with firm size. When occupational information was missing for this reason, the previous occupation registered was used if the difference in income was less than 10%, or coded these observations as missing. The population consists of those who had an occupation registered in 1996 and who were 65 years or younger. Individuals were censored when they reached age 66.

*Origin class* was defined as the occupational class in 1996 and is a time-constant covariate. The social class measure is based on the Erikson-Goldthorpe-Portocarero (EGP) occupational schema and coded according to the European Socioeconomic Classification (EseC) [39]. Due to lack of data on self-employed and small employers the measure contained seven categories instead of nine, which were combined into four more stringently graded social class groups (from high [1] to low [4]): [1] EseC 1: large employers; professional, administrative and managerial occupations; higher grade technician and supervisory occupations, [2] Esec 2: intermediate occupations and lower supervisory or lower technician occupations, [3] EseC 3: lower services, sales and clerical occupations, [4] EseC 4: lower technical occupations and routine occupations. *Destination class* is categorized each year from 1997 to 2012 and includes the four occupational class categories, as well as four others that include [1] not employed (including retired or inactive), [2] studying, [3] missing information, or [4] unemployed, which was identified through not being classified in an occupational class and having received unemployment benefits that year. Social mobility is categorized as: [1] upward mobility: destination class is higher than origin class [2]; downward mobility: destination class is lower than origin class [3]; not mobile: destination and origin class are the same [4]; not applicable, which overlaps with the statuses in the destination class category of not employed, unemployed, studying or having missing information.

Each individual is categorized according to whether they had any sickness absence paid by the Swedish Social Insurance Agency. According to Swedish legislation sickness absence, or sick leave, begins the second or third week of an illness, depending on the year and changes in legislation. Shorter sick leaves such as those due to the flu are not accounted for in this measure. After a sickness absence of seven days, a doctor’s note must be provided that verifies a disease or disorder that reduces the individuals’ work ability in order to receive sickness benefit payment by the social insurance system. Individuals may have multiple short term sick leaves (e.g., for one week) and be considered as having no sickness absence as no days were paid for by the Swedish Social Insurance Agency. The measure also does not allow us to know how many sick leaves are included in the total number of days paid on sick leave. Based on the sex- and year-specific distribution of days taken, quartiles were created to remove the effect of changes in legislation. Men and women are categorized as taking 0 days of sickness absence or in the bottom 25% of days taken (Q1), 25–50% (Q2), 50–75% (Q3), top 25% (Q4). The quartile measure of sickness absence allowed a non-linear and nuanced measure of poor health that provided the best model fit: Akaike and Bayesian Information Criteria values were at their minimum when comparing the baseline model using the quartile measure of sickness absence rather than other measures (continuous number of days or a dummy variable for having taken sickness absence).

## Methods

Discrete time event-history analysis [40] is used to examine the relationship between social mobility and mortality. This is a generalized linear model that is an extension of the piece-wise constant proportional hazards model where the odds ratios for mortality are conditional upon having survived up to that point in time. Time is treated as a discrete factor with one parameter for each segment of time, hereby designated with dummies for five-year age groups. In other words, the outcome is the odds of death at a given age conditional on the event not having occurred yet and compared to having the event at a later time. The model is specified as follows

$$ \log \left(\frac{P_{it}}{1-{P}_{it}}\right)=\alpha {D}_{it}+\beta {C}_{it}+\beta {V}_{it} $$

where *P*_*it*_ is the probability of death during interval *t. D*_*it*_ is a vector of dummies for age groups defined in five-year intervals, which serve as the baseline risk with coefficients *α*. *C*_*it*_ is a vector of covariates that are constant over time and *V*_*it*_ is a vector of covariates that vary over time, with coefficients *β*. All models were estimated with Stata version 15.

In the baseline model, the relationship between social mobility and mortality is adjusted for the time-constant variable of foreign-born status as well as the time-varying covariates of educational enrolment and attainment, marital status, and residence in metropolitan area. These covariates aim to capture the confounding influence of human capital, resources, background and partnership characteristics that have well established links to mortality and potentially to social mobility. In the second model, sickness absence in the previous year (entered into the model as a leading variable) is added to observe whether this potential antecedent explains the relationship between social mobility and mortality. If sickness absence leads to/prohibits mobility experiences that are relevant to mortality, this alignment would allow sickness absence to absorb the relationship between social mobility and mortality. Individuals are censored in our data if they emigrate, die, turn age 66, or when the end of the study period is reached.

The relationship between social mobility and mortality is assessed net of accumulated advantages and disadvantages by controlling for origin and destination status. Due to concerns that the origin and destination status effects would be biased by the mobile individuals and this would not allow a clean estimation of the mobility “effect”, the diagonal reference model (DRM) has been argued to be the most suitable estimator [41, 42]. In this study, the simpler strategy of controlling for origin and destination status is chosen, which better accommodates time-varying class indicators that include episodes of non-labor market participation and performed very similarly in a comparison with the DRM on the same data [43].

## Results

Table 1 shows descriptive statistics of our sample: the first column shows the total distribution over the covariates for men and women separately and the following columns show row percentages of person/years without sickness absence and with sickness absence recorded in quartiles. The *p*-value for a chi-square test of independence between sickness absence and each covariate is recorded in the final columns. As evident, taking sick leave and the extent of sick leave is statistically related to each of the covariates included in this analysis. Overall, sickness absence is higher among women than men and highest amongst those in older ages, with a prior marriage/registered partnership, foreign born, with lower education (<=2 years of secondary education), and who live in a rural or small city. Additionally, for both the origin and destination social class variables, a social gradient appears in which sickness absence is higher in lower social classes. Those who were studying generally had a similar incidence of having been on sick leave the previous year to those who were working, except for a higher frequency of very long sick leaves. This indicates a potential pattern in which individuals who have had long-term illnesses may be more prone to going back to school for re-training. The category “no activity” also has a high share of sickness absence in the destination class variable, as well as the unemployed category and “missing”, which includes employment in a smaller private firm and self-employment. These three categories largely make up the “not applicable” category in the social mobility variable, which correspondingly shows high sickness absence. Whereas we see that short and medium bouts of sick leave in the previous year are most frequent among the unemployed individuals, the longest sick leaves are most frequent for those who subsequently become inactive. In general, these patterns reflect what we would expect to see if those who stay in employment are healthier than those who exit. Relative to those who are not mobile, the share of sickness absence in the previous year is greatest for those who are downwardly mobile, and lowest among upwardly mobile individuals. Experiencing prolonged sickness absence was the rarest among very young individuals and for men and women who were upwardly mobile; only 0.92% of men and 2% of women who were upwardly mobile were in the highest quartile of sickness absence the year before.

**Table 1:** Descriptive statistics among men and women with and without registered sickness absence (SA, leading variable): Row percent (%) of person/years in each background variable category. SA measured in quartiles (Q1-Q4)

	Men (n men/years = 11,409,426)							Women (n women/years = 15,768,800)						
	Row % of all person/years	No SA	Q1 SA	Q2 SA	Q3 SA	Q4 SA	Chi2 *p*-value	Row % of all person/years	No SA	Q1 SA	Q2 SA	Q3 SA	Q4 SA	Chi2 *p*-value
Age														
17–25	1.6	95.0	1.7	1.5	1.3	0.6		1.5	91.3	3.4	2.4	2.0	0.9	
26–30	4.7	93.7	2.1	1.9	1.5	0.9		4.1	84.4	5.6	4.5	3.7	1.8	
31–35	9.1	92.9	2.4	2.0	1.6	1.2		7.9	82.2	6.0	4.9	4.2	2.8	
36–40	12.4	92.1	2.5	2.1	1.8	1.5		11.1	83.0	5.0	4.4	4.1	3.5	
41–45	14.1	91.6	2.5	2.2	2.0	1.7		13.5	84.0	4.4	4.0	3.9	3.8	
46–50	14.7	90.7	2.6	2.3	2.3	2.2		15.3	83.1	4.3	4.2	4.1	4.2	
51–55	15.7	89.2	2.7	2.6	2.7	2.8		16.8	81.2	4.6	4.6	4.7	5.0	
56–60	15.2	87.0	2.9	3.0	3.4	3.8		16.3	79.3	4.7	4.8	5.3	5.9	
61–65	12.6	88.1	2.2	2.6	3.2	3.9	0.0000	13.5	82.1	3.6	4.0	4.8	5.5	0.0000
Marital status														
Married/reg. Partnership	54.1	90.7	2.4	2.3	2.3	2.3		56.1	82.8	4.4	4.3	4.3	4.2	
Unmarried	33.2	90.9	2.5	2.3	2.2	2.0		25.9	83.8	4.7	4.1	3.9	3.5	
Prior marriage/reg. Partnership	12.8	86.9	3.0	3.1	3.3	3.7	0.0000	18.0	78.2	4.9	5.2	5.6	6.1	0.0000
Country of birth														
Born in Sweden	92.0	90.6	2.5	2.3	2.3	2.3		91.6	82.5	4.6	4.3	4.4	4.2	
Foreign born	8.0	86.6	3.1	3.0	3.2	4.1	0.0000	8.4	79.2	4.8	4.9	5.1	6.1	0.0000
Education														
In education	1.6	89.5	2.5	2.4	2.8	2.9		3.1	81.7	4.8	4.3	4.6	4.6	
< =2 years of sec. Education	46.0	87.3	3.3	3.1	3.1	3.3		47.3	79.6	5.0	4.9	5.0	5.5	
> 2 years of sec. Education	15.4	91.3	2.4	2.2	2.1	2.0		11.6	83.7	4.9	4.3	3.9	3.3	
< =3 years higher education	15.4	92.7	2.0	1.8	1.9	1.7		16.8	83.9	4.3	4.1	4.1	3.6	
> 3 years higher education	21.4	94.4	1.5	1.4	1.5	1.3	0.0000	21.1	86.2	3.6	3.6	3.6	3.0	0.0000
Residence														
Rural or small city	69.4	89.9	2.6	2.5	2.5	2.5		69.7	81.9	4.6	4.4	4.5	4.6	
Large city	30.6	91.1	2.4	2.2	2.2	2.2	0.0000	30.3	83.1	4.5	4.3	4.2	4.0	0.0000
Origin social class														
Professional	37.0	93.3	1.7	1.7	1.8	1.6		29.0	85.5	3.7	3.7	3.8	3.3	
Intermediate	11.2	93.0	1.8	1.7	1.8	1.7		20.2	84.5	4.2	3.9	3.9	3.6	
White collar workers	9.7	89.1	2.9	2.7	2.7	2.7		35.7	79.9	5.2	5.0	4.9	5.1	
Blue collar workers	42.1	87.2	3.4	3.2	3.1	3.2	0.0000	15.1	78.5	5.3	5.1	5.2	5.9	0.0000
Destination class														
Professional	29.6	94.3	1.7	1.6	1.5	1.0		24.3	86.9	3.8	3.7	3.4	2.2	
Intermediate	6.9	93.6	1.9	1.8	1.7	1.1		12.9	85.3	4.5	4.0	3.7	2.4	
White collar workers	5.5	89.4	3.3	3.0	2.7	1.7		20.0	81.7	5.8	5.1	4.5	2.9	
Blue collar workers	23.3	88.1	3.8	3.4	2.9	1.8		7.6	79.2	6.3	5.7	5.2	3.7	
Studying	1.2	90.8	1.9	1.8	2.4	3.2		2.3	82.4	4.0	3.9	4.5	5.2	
No activity	4.7	85.2	1.3	1.7	3.4	8.4		5.2	81.0	1.1	2.2	4.6	11.1	
Unemployment	3.9	84.1	4.4	4.2	4.2	3.0		3.9	76.8	7.2	6.1	5.8	4.1	
Missing	24.9	88.7	2.3	2.4	2.7	3.9	0.0000	23.9	78.5	4.2	4.5	5.2	7.7	0.0000
Social mobility														
Upward	7.5	93.5	2.1	1.8	1.6	0.9		7.0	85.9	4.7	4.0	3.4	2.0	
No mobility	54.6	91.4	2.7	2.4	2.2	1.4		55.1	83.9	4.9	4.5	4.1	2.7	
Downward	3.2	90.7	2.8	2.6	2.4	1.6		2.7	82.5	5.4	4.8	4.4	3.0	
Not applicable	34.7	87.8	2.4	2.5	3.0	4.4	0.0000	35.2	78.9	4.1	4.3	5.1	7.6	0.0000

Note: missing categories for marital status and education not shown as shares were < =0.1%

Table 2 displays descriptive statistics for deaths in the sample and how deaths are distributed across levels of sickness absence in the previous year. For all-cause mortality, 0.22 and 0.16% of person/years ended in death for men and women, respectively, whereas these shares were 0.09 and 0.10% for cancer-related mortality, 0.07 and 0.02% for CVD-related mortality and 0.02 and 0.01% for suicide. When men and women had not had sickness absence in the previous year, the shares of all-cause mortality dropped to 0.18 and 0.11%, respectively, and increased to 1.21 and 0.80% when sickness absence had been among the highest quartile in the preceding year. A gradient appears for both men and women across quartiles of sickness absence across the causes of death in this study. The only exception is CVD-related mortality and suicide for women, in which little or no change in mortality is observed until the top two quartiles of sickness absence. The *p*-value for a chi-square test of independence once again shows a bivariate relationship; sickness absence is statistically linked to all-cause mortality as well as the three specific causes of death. These patterns support the use of sickness absence as a measure of health that is linked to mortality.

**Table 2:** Men’s and women’s mortality by sickness absence (SA, leading variable measured in quartiles): Percent of all person/years

	All mortality	Cancer-related mortality	CVD-related mortality	Suicide
Men	0.22	0.09	0.07	0.02
N deaths	25,351	9724	7570	1917
No SA	0.18	0.06	0.06	0.01
< 25% SA	0.29	0.11	0.08	0.02
25–50% SA	0.41	0.17	0.10	0.04
50–75% SA	0.63	0.30	0.14	0.05
> 75% SA	1.21	0.60	0.26	0.07
chi2 *p*-value	0.0000	0.0000	0.0000	0.0000
Women	0.16	0.10	0.02	0.01
N deaths	24,772	15,826	3652	1097
No SA	0.11	0.07	0.02	0.01
< 25% SA	0.13	0.08	0.02	0.01
25–50% SA	0.19	0.12	0.02	0.01
50–75% SA	0.33	0.23	0.04	0.02
> 75% SA	0.80	0.61	0.06	0.03
chi2 *p*-value	0.0000	0.0000	0.0000	0.0000

**Fig. 1:**
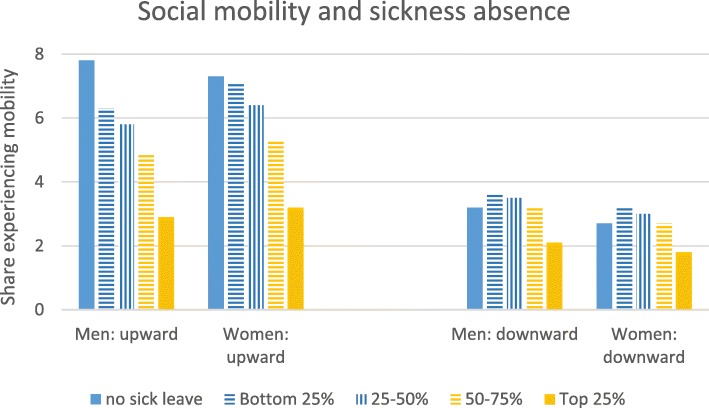
Social mobility and sickness absence in prior year: share of person/years

Figure 1 shows two patterns in how social mobility is linked to sickness absence descriptively. First, upward mobility is negatively related to sick leave length. The difference between no sick leave and any sick leave is greatest for men who were upwardly mobile, suggesting they have the greatest returns to good health and that upward mobility for women may involve less selection. Second, downward mobility has an inverted u-shape relationship to previous sick leave length and this occurs similarly for men and women. Downward mobility is more frequent for those who have been on short or moderate length sick leave than no leave at all, but downward mobility is even less frequent for those who have been on long sick leaves—likely due to the higher likelihood that they subsequently became inactive.

Table 3 shows selected results from discrete time hazard regressions on the relationship between intra-generational social mobility and mortality. The first column provides the baseline estimates that are adjusted for a set of control variables, whereas the second column provides estimates when also adjusted for sickness absence in the previous year. The estimates for downward and upward mobility are in reference to being non-mobile, unemployment estimates are in reference to blue collar working class positions and estimates for the quartile location in the distribution of sick leave length in the previous year is in reference to not having been on sick leave.

**Table 3:** Discrete time hazard model (selected) results for all-cause mortality, cancer-related mortality and suicide, with and without sickness absence

	All-cause mortality		Cancer-related mortality		CVD-related mortality		Suicide	
	Adjusted baseline	Including sickness absence	Adjusted baseline	Including sickness absence	Adjusted baseline	Including sickness absence	Adjusted baseline	Including sickness absence
Men								
Social mobility								
No mobility	1	1	1	1	1	1	1	1
Upward	0.77 (0.71–0.84)	0.82 (0.76–0.89)	0.78 (0.68–0.89)	0.85 (0.74–0.97)	0.74 (0.64–0.85)	0.76 (0.66–0.88)	0.76 (0.59–0.99)	0.80 (0.62–1.04)
Downward	1.06 (0.97–1.16)	1.01 (0.93–1.11)	1.23 (1.07–1.42)	1.16 (1.00–1.33)	0.99 (0.84–1.16)	0.96 (0.82–1.13)	0.74 (0.52–1.05)	0.72 (0.51–1.01)
Unemployed during the year								
Relative to blue collar working class	1.67 (1.56–1.78)	1.53 (1.43–1.64)	1.56 (1.38–1.77)	1.40 (1.23–1.58)	1.44 (1.28–1.63)	1.38 (1.23–1.56)	1.85 (1.49–2.29)	1.67 (1.34–2.07)
Sickness absence								
none		1		1		1		1
Bottom 25%		1.65 (1.54–1.77)		1.84 (1.64–2.06)		1.38 (1.21–1.57)		1.66 (1.31–2.11)
25–50%		2.04 (1.92–2.17)		2.55 (2.33–2.80)		1.50 (1.33–1.69)		2.42 (1.98–2.97)
50–75%		2.68 (2.55–2.81)		3.71 (3.45–3.99)		1.68 (1.51–1.87)		2.88 (2.41–3.45)
Top 25%		3.81 (3.67–3.96)		5.51 (5.21–5.83)		2.42 (2.24–2.62)		3.35 (2.86–3.92)
Number of observations	11,409,426	11,409,426	11,409,426	11,409,426	11,409,426	11,409,426	11,409,426	11,409,426
Log likelihood	− 167,177,7	− 164,800,1	−72,620,6	−70,906,4	−58,102,8	−57,862,8	−18,189,4	−18,036,5
Women								
Social mobility								
No mobility	1	1	1	1	1	1	1	1
Upward	0.98 (0.90–1.06)	1.03 (0.93–1.14)	1.08 (0.97–1.19)	1.15 (1.04–1.28)	0.84 (0.68–1.04)	0.86 (0.69–1.06)	0.65 (0.43–0.96)	0.67 (0.45–1.00)
Downward	1.08 (0.97–1.19)	1.03 (0.95–1.12)	1.14 (1.01–1.30)	1.08 (0.95–1.23)	1.05 (0.82–1.33)	1.03 (0.81–1.31)	0.95 (0.60–1.51)	0.92 (0.58–1.46)
Unemployed during the year								
Relative to blue collar working class	1.66 (1.51–1.82)	1.51 (1.43–1.64)	1.76 (1.56–2.00)	1.57 (1.38–1.78)	1.20 (0.97–1.49)	1.17 (0.94–1.45)	1.55 (1.03–2.33)	1.40 (0.93–2.11)
Sickness absence								
none		1		1		1		1
Bottom 25%		1.38 (1.29–1.47)		1.41 (1.30–1.54)		1.25 (1.06–1.47)		1.65 (1.26–2.17)
25–50%		1.78 (1.68–1.88)		1.98 (1.84–2.12)		1.26 (1.08–1.47)		2.46 (1.96–3.09)
50–75%		2.64 (2.53–2.76)		3.12 (2.96–3.30)		1.52 (1.34–1.74)		2.79 (2.27–3.42)
Top 25%		4.72 (4.57–4.87)		6.23 (6.00–6.47)		1.93 (1.74–2.15)		4.06 (3.45–4.79)
Number of observations	15,769,783	15,768,783	15,769,783	15,769,783	15,769,783	15,769,783	15,769,783	15,769,783
Log likelihood	− 172,335,5	− 168,331,5	−116,915,7	−113,083,5	−31,983,7	−31,904,4	−11,283,3	−11,133,8

Note: results adjusted for age, marital status, foreign born, educational attainment, living in metropolitan area, and origin and destination status. Confidence intervals in parentheses

The indicator for poor health, sickness absence in the previous year, was consistently related to all-cause mortality and the three specific causes of mortality, net of other individual characteristics controlled for in the model. Previous poor health is most linked to cancer-related mortality for both men and women, followed by suicide. It was the least linked to CVD-related mortality. The increase in the odds of mortality at each higher quartile of sick leave length was also steepest for cancer-related mortality. However, the highest odds of mortality associated with taking only a short sickness absence (Q1) were found for suicide for women and not cancer-related mortality.

### All-cause mortality

Excess death due to all causes was lower (OR 0.77, 95% CI 0.71–0.84) when men had been upwardly mobile, but upward mobility was not linked to all-cause mortality for women and downward mobility was not statistically related to all-cause mortality for either men or women. Unemployment increased the odds of all-cause mortality by 1.67 (95% CI 1.56–1.78) for men and 1.66 (95% CI 1.51–1.82) for women, relative to being employed in a blue-collar working class occupation. When adjusting for sickness absence in the relationship between upward mobility and mortality for men, the odds of death declined from being 23% lower than the non-mobile to 18% lower. The odds of death for both men and women declined in relation to being unemployed to 53% higher for men and 51% higher for women when accounting for sick leave.

### Cancer-related mortality

All-cause cancer mortality was associated with being downwardly mobile among men and women both, respectively, compared to not being mobile (Table 3: OR 1.23, 95% CI 1.07–1.42; OR 1.14, 95% CI 1.01–1.30). When adjusting for prior sickness absence, this association was reduced among men (OR 1.16, 95% CI 1.00–1.33) and women (OR 1.08, 95% CI 0.95–1.23): the difference between not being mobile and being downwardly mobile became no longer statistically significant among women. Among men, upward mobility was associated with lowered odds of death due to cancer (OR 0.78, 95% CI 0.68–0.89), but the lower odds lessened from 0.78 to 0.85 when controlling for sickness absence. No relationship between upward mobility and cancer-related mortality appeared for women in the baseline model, but upward mobility was linked to higher odds of cancer mortality when adjusting for sickness absence (OR 1.15, 95% CI 1.04–1.28). That mortality is higher for upwardly mobile women is likely linked to the predominance of breast cancer related deaths and its unusual positive class gradient. The difference related to accounting for having been on sick leave may be linked to early detection and how this intersects with class and mobility. Unemployment also remained positively associated with cancer-related mortality when accounting for sickness absence (OR 1.40 for men, 95% CI 1.23–1.58; OR 1.57 for women, 95% CI 1.38–1.78).

### CVD-related mortality

Neither social mobility nor unemployment were linked to CVD-related mortality for women. For men, upward mobility was negatively associated with CVD-related mortality, both before and after adjusting for sickness absence (OR 0.76, 95% CI 0.66–0.88) and the difference between the two estimates appeared to be negligible. Similarly, the estimates related to unemployment remained mostly stable when including sickness absence in the model for men (OR 1.38, 95% CI 1.23–1.56).

### Suicide

Table 3 shows that upward mobility was associated with lowered odds of suicide in men and women, respectively (OR 0.76, 95% CI 0.59–0.99; OR 0.65, 95% CI 0.43–0.96). These associations were no longer statistically significant when adjusting for sickness absence. Downward mobility was not associated with suicide for men or women. Odds ratios for suicide were elevated when men and women, respectively, had been unemployed (OR 1.85, 95% CI 1.49–2.29; OR 1.55, 95% CI 1.03–2.33), but this association disappeared for women when sickness absence in the previous year was controlled for (OR 1.40, 95% CI 0.93–2.11).

### Average marginal effects

**Fig. 2:**
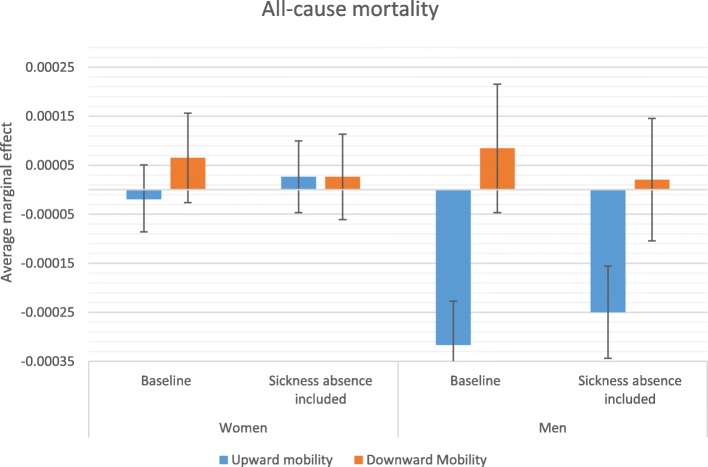
Average marginal effects in relation to no mobility on all-cause mortality

**Fig. 3:**
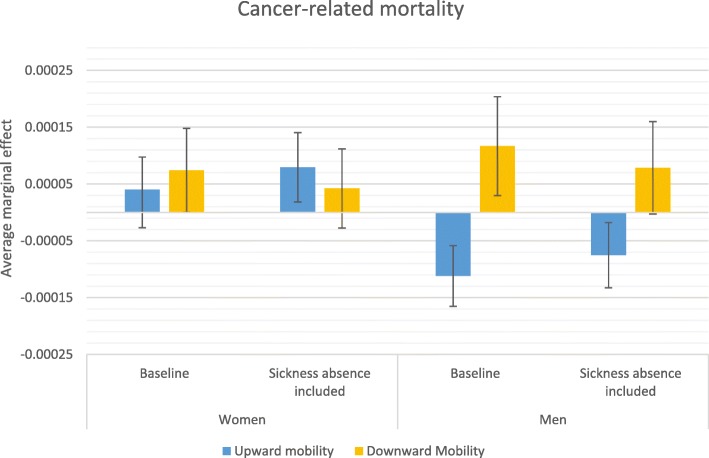
Average marginal effects in relation to no mobility on cancer-related mortality

**Fig. 4:**
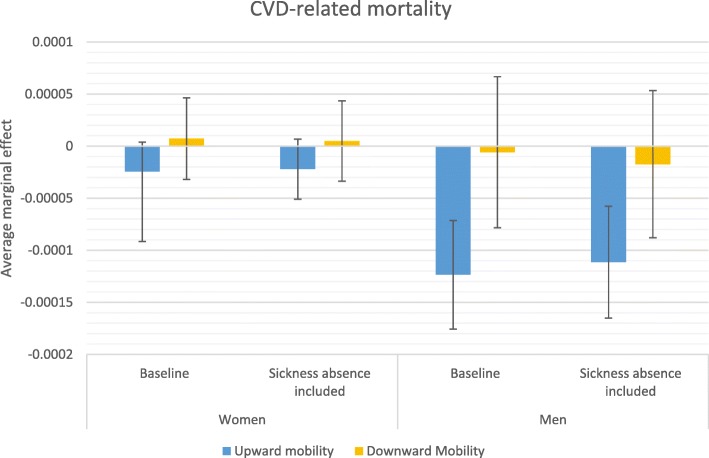
Average marginal effects in relation to no mobility on CVD-related mortality

**Fig. 5:**
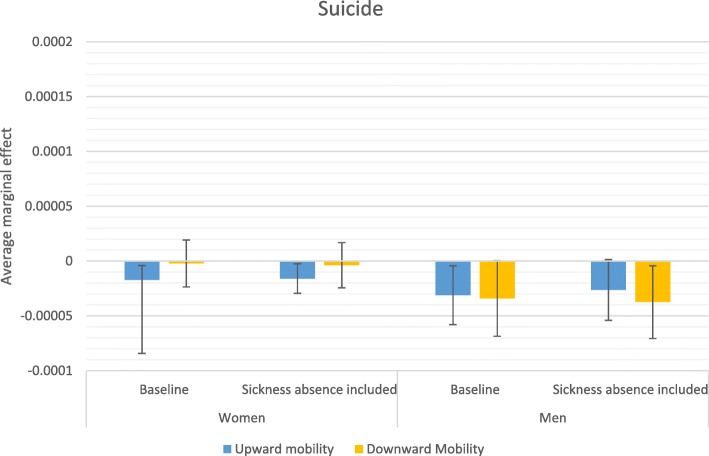
Average marginal effects in relation to no mobility on suicide

Stepwise comparison of odds ratios—i.e., comparing odds ratios from two models in which an additional variable is introduced in the second model—may be inaccurate due to changes in omitted variable bias created by including an additional variable [44]; therefore, more careful conclusions about health selection are drawn from a comparison of average marginal effects (AME) estimated from the baseline model and from the model that accounts for sickness absence in the previous year. AMEs are also useful because they give more substantive information on how a factor matters; they reflect the average influence of the situation that actually exists in the population. Figures 2, 3, 4 and 5 display how the AMEs (the difference in predictive margins between the non-mobile and the mobile) change when accounting for prior sickness absences. AMEs are calculated for all those who were observed in an occupational class. If health selection into mobility and mortality were playing a role in the social mobility and mortality relationship, we would expect to see the average marginal effect become either statistically insignificant or shift toward 0 when accounting for sickness absence, which would mean that the difference between the non-mobile and the mobile disappears or lessens.

The predicted relationship between social mobility and mortality is largely the same as the pattern observed for the previous odds ratios. For all-cause mortality (Fig. 2) and CVD-related mortality (Fig. 4), the only significant relationship appears for men’s upward mobility and this relationship remains even after adjusting for previous sickness absence. Figure 3 displays the same finding for cancer-related mortality for men, where the lowered mortality related to upward mobility was weakened by controlling for previous sick leave, but remained statistically distinct from the predicted mortality of the non-mobile. The predicted mortality of the downwardly mobile became statistically indistinct, however, in the average marginal effects for both men and women.

In relation to suicide, the influence of upward mobility remained distinct from non-mobility for women even when adjusting for sickness absence. In contrast to the mobility patterns for most other causes of death, being both upwardly and downwardly mobile is related to lower mortality in men than for the non-mobile. Adjusting for sickness absence alters the predicted influence of upward mobility for men in the expected direction of weakening the relationship, but removing the potential influence of illness leading to downward mobility slightly strengthens the lower predicted mortality associated with downward mobility. Removing the influence of illness appears to therefore potentially increase the selectivity of those who are downwardly mobile toward being less at risk of suicide.

**Fig. 6:**
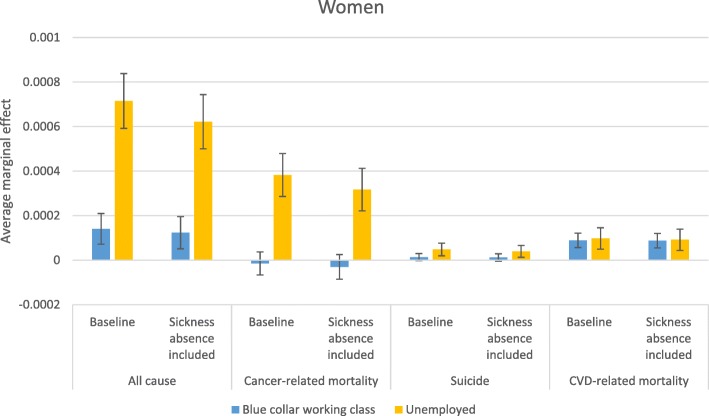
Average marginal effects in relation to working in the professional class, women

**Fig. 7:**
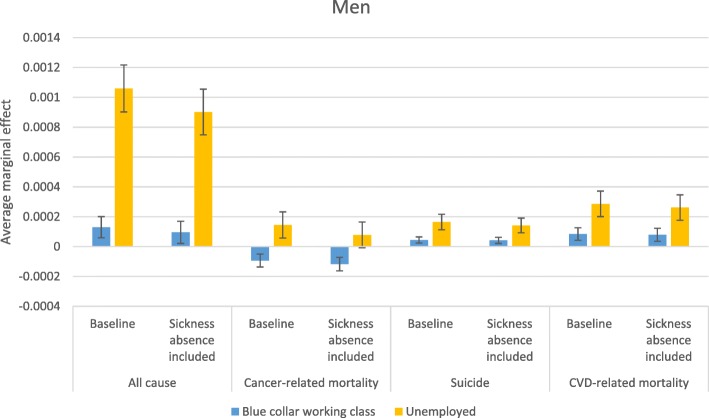
Average marginal effects in relation to working in the professional class, men

The changes due to controlling for sickness absence in the predicted mortality related to unemployment are shown in Figs. 6 and 7 for women and men, respectively. In these figures, the reference group is the professional class in order to show how both the lowest class (blue collar working class) and unemployment compare. In addition, the social mobility and origin status variables were not kept in these models due to the inability to estimate AMEs of unemployment with multiple overlapping categories; dropping these two variables did not change the odds ratios for unemployment. In relation to the professional class, unemployment was related to every cause of death for men and women and adjusting for sickness absence did not absorb the relationship with any cause of death except for cancer for men. The predicted influence on mortality was weakened when controlling for previous sickness absence for women’s cancer-related mortality and for all-cause mortality for both men and women. However, the comparison between unemployment and blue collar working class positions shows that unemployed men are still at a higher risk of cancer-related mortality, and the difference due to a change in reference groups likely reflects the higher selection out of the labor market for those with cancer who previously worked in blue class working collar jobs. The predicted mortality due to CVD and suicide did not differ between blue collar working class and unemployed women before or after including sickness absence.

## Discussion

The aim of this study was to examine how poor health intersects with social mortality and its relationship to mortality. If recent health in terms of sickness absence explains the short-term association between intragenerational social mobility and mortality then we can conclude health selection plays a role in how individuals are sorted into classes and in the mortality gradient. Descriptive statistics indicate health selection both out of the labor force and into mobility: Among men and women, a higher share of prior sickness absence was associated with inactivity, downward mobility and unemployment, and lower levels of sickness absence were evident among upwardly mobile compared to non-mobile states. For each cause of death in men and women, mortality is also highest when there has been heavy use of sickness absence.

The results from multivariate discrete time hazard analyses present similar patterns when looking at all-cause mortality and CVD-related mortality, which is not unexpected given that cardiovascular diseases are the leading cause of death in Sweden for men and women. When predicting mortality differences, the results for both all-cause and CVD-related mortality suggest only minor health selection effects in the relationship between upward mobility and mortality. Men appear to be less at risk of CVD-related mortality when they have been upwardly mobile and this lower risk is only moderately altered when accounting for sick leave. A similar pattern appears for other causes of death, where previous poor health weakens but does not explain the lower cancer-related mortality of men and lower suicide mortality of women who have been upwardly mobile. This implies that other causal or indirect selection mechanisms may play a role in this relationship, potentially including well-being benefits of having achieved a higher class status or characteristics of an individual that lead to less career advancement and more cancer and CVD-related mortality for men, suicide for women. No research to date offers evidence on how positive changes in well-being through upward mobility might influence mortality positively. Because estimates in this study are net of origin and destination effects, the advantages and disadvantages of class position such as income and social prestige as well as work environment characteristics should not play a role in the relationship. How upward mobility may offer protection from premature mortality is a question to answer in future research.

In contrast, previous poor health weakens the relationship between upward mobility and suicide for men. To the extent that the relationship is explained by prior health and selection, it may be that individuals choose not to search for higher job positions; alternatively, they may not be selected for promotion by an employer due to stigma related to the disorder, or not hired for a new position due to lower productivity that may result from having been on sick leave. These pathways do not seem to be the same for women, as upward mobility was rarely linked to lower mortality for women. This confirms the descriptive finding that upward mobility and sickness absence were less related for women than men; we know that women face different obstacles in reaching higher social class positions than men [45] and health appears potentially more important to men’s upward mobility prospects than women’s.

Having previously been in poor health and taken sick leave from work does largely explain the relationship between downward mobility and cancer mortality for both women and men. Chronic diseases such as cancer were expected to be the most likely to create health selection into social mobility and mortality since cancer mortality should be preceded by a period of time in poor health long enough to affect a career. Although suicide may be thought to be preceded by psychiatric disorders that potentially influences social mobility chances [33] as well, previous studies have found little support for selection effects within the labor market [4, 9, 10, 14, 17, 18]. Psychiatric health selection effects may be more evident in the pathway out of the labor market, particularly for those individuals with very severe psychiatric disorder such as schizophrenia [9, 17].

No earlier study has examined health selection of cancer disease into social mobility or by using cancer-related death as an outcome. The scenario that is indicated by the findings in this study is that individuals with cancer often continue to work but change jobs to a lower occupational class, perhaps to lessen work duties and responsibilities because of limited capacity. Due to the Swedish context, where regulation surrounding sickness absence protects workers’ positions, we might suspect this is a process solely resulting from individuals’ preferences. Unemployment, however, is less likely to be the result of an individual’s choice, healthy or in poor health. That previous sickness absence weakens the relationship between unemployment and all-cause and cancer-related mortality indicates that Swedish legislation may not be offering enough job protection for those in poor health, who are not able to leave the labor market completely but also cannot find a new job and begin receiving unemployment benefits instead. More research is needed to explore whether employers are discriminating against those who have been on sick leave when they return to work.

### Methodological considerations

Since the occupational class data only include a random sample of those persons working in smaller private firms and no individuals who become self-employed, the observations not covered by that data are coded as missing and they are not contributing to the social mobility estimates; the results cannot be generalized to individuals in very small firms or those self-employed. The sickness absence measure also comes with a few limitations. First, we do not know how many episodes of illness a person had during a year. For those who had multiple episodes, the total number of days spent ill is underestimated because the employer paid days are uncounted for each sickness leave spell in our data. Second, sickness absence, poor health and diagnosed illnesses do not perfectly overlap [46] and we can expect that sickness absence can sometimes be for common conditions that are not fatal such as back pain. Nevertheless, our results show a strong mortality gradient associated with the measure of sickness absence, confirming that the measure conveys either the extent of illness or an overall health potential that is related to mortality.

## Conclusions

Across the causes of death studied here, upward mobility appears to be the main pathway through which social mobility is linked to mortality in Sweden. Better longevity for upwardly mobile men largely persists after adjusting for previous poor health. However, the overall conclusion of this study is that health selection accounts for a small part of why upward mobility is associated with lower mortality and plays a more definitive role in how downward mobility is linked to cancer related deaths. Using an analytical strategy that does not preclude the role of direct health, individuals in Sweden therefore appear to be sorted into occupational trajectories according to health. That health selection plays any role in how social mobility and mortality are related in Sweden may be unexpected in a context with strong job protection. Job protection does not, however, equalize opportunities for upward mobility, which may be reduced for those who have been ill. But because intragenerational upward mobility and mortality remained related after adjusting for an objective and broad measure of poor health, the findings of this study point to other important pathways to explore through which social mobility and mortality are linked, such as indirect selection or social causation.

## Data Availability

All relevant data is owned by Statistics Sweden and is available upon request by interested researchers who apply and are granted access to the data. Data are available from Statistics Sweden https://www.scb.se/en/ for researchers who meet the criteria for access to confidential data. The authors confirm they had no special access or privileges and that other researchers may access the data in the same way.

## Abbreviations

AME: Average marginal effect
CI: Confidence interval
CVD: Cardiovascular disease
DRM: Diagonal reference model
EGP: Erikson-Goldthorpe-Portocarero
EseC: European Socioeconomic Classification
ICD-10: International classification of diseases and related health problems, 10th revision
OR: Odds ratio
